# Antibiotic Discovery: Where Have We Come from, Where Do We Go?

**DOI:** 10.3390/antibiotics8020045

**Published:** 2019-04-24

**Authors:** Bernardo Ribeiro da Cunha, Luís P. Fonseca, Cecília R. C. Calado

**Affiliations:** 1 Institute for Bioengineering and Biosciences (IBB), Instituto Superior Técnico (IST), Universidade de Lisboa (UL); Av. Rovisco Pais, 1049-001 Lisboa, Portugal; luis.fonseca@tecnico.ulisboa.pt; 2 Departamento de Engenharia Química, Instituto Superior de Engenharia de Lisboa (ISEL), Instituto Politécnico de Lisboa (IPL); R. Conselheiro Emídio Navarro 1, 1959-007 Lisboa, Portugal; ccalado@deq.isel.pt

**Keywords:** antibiotic discovery platforms, drug screening, semi-synthesis, fully synthetic antibiotics, genomics, proteomics, metabolomics, lipidomics, metagenomics

## Abstract

Given the increase in antibiotic-resistant bacteria, alongside the alarmingly low rate of newly approved antibiotics for clinical usage, we are on the verge of not having effective treatments for many common infectious diseases. Historically, antibiotic discovery has been crucial in outpacing resistance and success is closely related to systematic procedures—platforms—that have catalyzed the antibiotic golden age, namely the Waksman platform, followed by the platforms of semi-synthesis and fully synthetic antibiotics. Said platforms resulted in the major antibiotic classes: aminoglycosides, amphenicols, ansamycins, beta-lactams, lipopeptides, diaminopyrimidines, fosfomycins, imidazoles, macrolides, oxazolidinones, streptogramins, polymyxins, sulphonamides, glycopeptides, quinolones and tetracyclines. During the genomics era came the target-based platform, mostly considered a failure due to limitations in translating drugs to the clinic. Therefore, cell-based platforms were re-instituted, and are still of the utmost importance in the fight against infectious diseases. Although the antibiotic pipeline is still lackluster, especially of new classes and novel mechanisms of action, in the post-genomic era, there is an increasingly large set of information available on microbial metabolism. The translation of such knowledge into novel platforms will hopefully result in the discovery of new and better therapeutics, which can sway the war on infectious diseases back in our favor.

## 1. Introduction—The Desperate Need for New Antibiotics

Infectious diseases have been a challenge throughout the ages. From 1347 to 1350, approximately one-third of Europe’s population perished to Bubonic plague. Advances in sanitary and hygienic conditions sufficed to control further plague outbreaks. However, these persisted as a recurrent public health issue. Likewise, infectious diseases in general remained the leading cause of death up to the early 1900s, e.g., accounting for 25% of England’s mortality. However, by the mid-1900s, the mortality of infectious diseases in England shrunk to under 1% after the commercialization of antibiotics [1], which given their impact on the fate of mankind, were regarded as a ‘medical miracle’. Moreover, the non-therapeutic application of antibiotics has also greatly affected humanity, for instance those used as livestock growth promoters to increase food production after World War II. 

The term ‘antibiotic’ was introduced by Selman Waksman as any small molecule, produced by a microbe, with antagonistic properties on the growth of other microbes [2]. An antibiotic interferes with bacterial survival via a specific Mode-Of-Action (MOA) but more importantly, at therapeutic concentrations, it is sufficiently potent to be effective against infection and simultaneously presents minimal toxicity. Most antibiotic classes in use today were identified in the 1940–1960s, a period referred to as the antibiotic golden age. During this period, it was common belief that, given the antibiotics discovered and particularly the rate at which they were discovered, infectious diseases would soon be a controlled public health issue [3,4]. In fact, in 1970, the US Surgeon General stated “It’s time to close the book on infectious diseases… and shift national resources to such chronic problems as cancer and heart disease” [5]. 

Currently, more than 2 million North Americans acquire infections associated with antibiotic resistance every year, resulting in 23,000 deaths [6]. In Europe, nearly 700 thousand cases of antibiotic-resistant infections directly develop into over 33,000 deaths yearly [7], with an estimated cost over €1.5 billion [8]. Despite a 36% increase in human use of antibiotics from 2000 to 2010 [9], approximately 20% of deaths worldwide are related to infectious diseases today [10]. This situation deteriorated further as nosocomial infections became a leading cause of morbidity and mortality [11], resulting in lengthier hospital stays and increased health care costs [12]. Furthermore, over 15% of nosocomial infections are already caused by multidrug-resistant pathogens [13]—for some of which, there are no effective antimicrobials [14]. Future perspectives are no brighter, for instance, a government commissioned study in the United Kingdom estimated 10 million deaths per year from antibiotic resistant infections by 2050 [15]. 

The increase in drug-resistant pathogens is a consequence of multiple factors, including but not limited to high rates of antimicrobial prescriptions, antibiotic mismanagement in the form of self-medication or interruption of therapy, and large-scale antibiotic use as growth promotors in livestock farming [16]. For example, 60% of the antibiotics sold to the USA food industry are also used as therapeutics in humans [17]. To further complicate matters, it is estimated that $200 million is required for a molecule to reach commercialization [18], with the risk of antimicrobial resistance rapidly developing, crippling its clinical application, or on the opposing end, a new antibiotic might be so effective it is only used as a last resort therapeutic, thus not widely commercialized. Either way, the bottom line implies similar risks with considerably lower returns on investment compared with other drugs [19], which renders antibiotic discovery an unattractive business. In an attempt to counter this scenario, the European Federation of Pharmaceutical Industries and Associations consorted with the European Union to establish the largest worldwide life sciences public–private partnership, the Innovative Medicines Initiative. Through funding and a highly ambitious agenda, under the New Drugs for Bad Bugs program, this initiative encourages action in areas ranging from antibiotic discovery, clinical research, through to reshaping the use of antibiotics, in hopes of catalyzing the approval of novel antibiotics [20].

**Figure 1:**
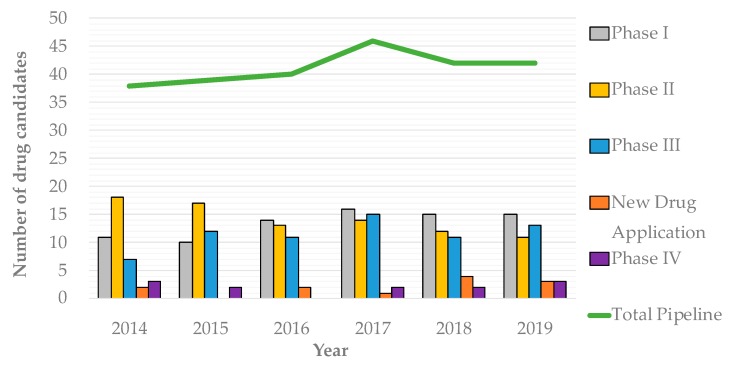
Evolution of the total antibiotic pipeline and the antibiotic pipeline by stage of development, which includes: Clinical Trials ranging from Phase I, to evaluate safety; Phase II, to access effectiveness and safety; Phase III, to gather statistically significant data on safety, effectiveness and benefits-versus-risk; submission of a New Drug Application, for marketing approval; and lastly, Phase IV for post-marketing surveillance.

The systematic procedures—Antibiotic Discovery Platforms (ADPs)—behind the discovery of major antibiotic classes, which fueled the antibiotic golden age, have become exhausted. Modern ADPs have yielded redundant discoveries and/or failed in translation to the clinic, which dimmed the overly optimistic expectations created with the development of novel technologies throughout the genomics era. From 2004–2009, the overall rate of antibacterial approval was a mindboggling single drug per year [21], which doubled from 2011–2014 when the FDA approved a still impressively scarce eight new antibiotics or combinatorial therapies [22]. According to the antibiotic pipeline surveillance by The Pew Charitable Trusts [23], from 2014 onwards, the situation is slowly improving, if at all. As seen on Figure 1, the total antibiotic pipeline appears to be timidly increasing, although the number of drug candidates close to approval (phase III clinical trials and those that have filed a New Drug Application) or recently approved (phase IV) remains alarmingly low. Despite great efforts, most approved antibiotics only target either the ribosome, cell wall synthesis machinery and DNA gyrase or topoisomerase [24,25]. Beyond conventional antibiotics, some interesting therapeutic alternatives are noteworthy, including bacteriophages, antivirulence strategies, probiotics, vaccines, immune stimulation, antimicrobial peptides, antibiofilm therapies and antibodies, among others. Despite some of these alternatives having reached clinical trials, it is estimated that across the next 10 years, over £1.5 billion will be needed to further test and develop them before their clinical impact is felt [26]. 

During 2011, the director general of the World Health Organization made the clear forewarning that we are currently “on the brink of losing these miracle cures… In the absence of urgent corrective and protective actions, the world is heading towards a post-antibiotic era, in which many common infections will no longer have a cure and, once again, kill unabated” [27]. Besides a more efficient management of antibiotic use, there is a pressing need for new platforms capable of consistently and efficiently delivering new lead substances, which should attend their precursors impressively low rates of success, in today’s increasing drug resistance scenario. The present manuscript reviews the discovery timeline of the major antibiotic classes from an ADPs perspective, highlighting their underlying technological basis and the context of their application, beginning with the birth of chemotherapy, the establishment of the Waksman platform, semi-synthesis and fully synthetic antibiotics, followed by the technological revolution during the genomics era, and the present-day efforts in the post-genomics era. 

## 2. The Birth of Antimicrobial Chemotherapy

Traditional behaviors and primitive rituals suggest ancient human use of antibiotics [28,29], although the first scientific record of the therapeutic use of antibiotics dates to 1899 when Emmerich and Löw explored the therapeutic potential of *Pseudomonas aeruginosa* extracts. While their investigation was discontinued due to inconsistent effects, the antimicrobial effect observed was later associated with quorum-sensing molecules [30]. When discussing antimicrobial chemotherapy, highlighting the contributions of Pasteur, Lister and Koch to the foundation of medical microbiology is a tribute of sorts, for which reviews are available [31,32,33,34]. The road towards the first modern antimicrobial began in 1854, when Antoine Béchamp produced aniline, via the reduction of nitrobenzene with iron in the presence of hydrochloric acid. In 1859, Béchamp produced atoxyl, by reacting aniline with arsenic acid, in his pursuit of developing aniline derivatives. Simultaneously, Paul Ehrlich noticed that chemical dyes stained specific histological and cellular structures, which inspired his side-chain theory in 1897, where he hypothesized about therapy targeting structures exclusive to pathogens [35]. Ehrlich, together with Alfred Bertheim and Sahachiro Hata, synthetized and screened multiple arsenical derivatives based on Béchamp’s discovery of atoxyl and, by 1907, discovered Salvarsan [36], the first antimicrobial that was an effective and safer therapeutic against syphilis, which became the most prescribed drug until the introduction of penicillin [37].

The systematic application of chemical modifications to expand a library of lead molecules, followed by screening its effect on a disease model contributed to the discovery of Neosalvarsan, a more water-soluble derivative with reduced side effects, and laid the foundation of modern pharmaceutical research. Given its success, the Friedrich Bayer Company explored synthetic chemicals for therapeutic purposes in the 1920s [38]. The azo compounds, a class of synthetic dyes with antibacterial activity, were the starting point for the synthesis of diverse structural variants. In 1932, Gerhard Domagk recognized the curative potential of Prontosil, synthetized by chemists Josef Klarer and Fritz Mietzsch, from studies on streptococci-infected mice and later, on two dire cases of children in life-threatening situations, including Domagk’s daughter. Prontosil became commercially available by 1935, simultaneous to the discovery of its active principle, which was unrelated to the azo functional group or the dye fraction. In fact, Prontosil is a precursor to the active molecule, sulfanilamide, widely used in the dye industry, hence not patentable, and whose synthesis was readily achievable. In the following years, over 5000 derivatives, known as the sulpha drugs, were synthetized, some of which are still used today, e.g., sulfamethoxazole. 

Arguably, it was Alexander’s Fleming ‘accidental’ detection of Staphylococci growth inhibition around mold colonies in petri dishes, forgotten at his lab throughout a holiday period, that mostly impacted the future of antimicrobial discovery [39]. Fleming’s observation in 1928 motivated his studies on the mold’s product, penicillin, regarding its activity spectrum, potency, leukocyte interaction and toxicity. In fact, it was the first substance noted to present more antibacterial than antileukocytic activity [40]. Fleming’s rigorous methods, and their underlying rational, are still hallmarks for antibiotic discovery. Nonetheless, Fleming faced problems associated with the large-scale growth of the penicillin-producing mold and it was not until 1939 that Howard Florey, Norman Heatly and Ernst Chain described a method that made penicillin sufficiently available for clinical testing. This bioprocess was greatly up-scaled when Florey and Heatly moved to the USA and Canada, given the necessity of antibiotics imposed by World War II [41]. Ultimately, their work on bioprocess optimization surpassed penicillin production, as it promoted the fermentation industry, which is highly relevant for the production of diverse antibiotics and other medicines such as insulin, erythropoietin, interferon, and antibodies, among others [42].

Although penicillin’s bioprocess scale-up breakthroughs enabled its widespread clinical use during the late period of World War II, efforts pursued an outperforming chemical synthesis protocol. During late 1945, penicillin antimicrobial activity was traced to the B-lactam ring [43]. Ernst Chain believed that fully synthetic penicillin would require new chemical techniques, achieved in 1950 by John Sheehan, from which the first synthetic natural penicillin V was produced in 1957. The year after, Sheehan described the production of 6-aminopenicillanic acid (6-APA) via both semi- and fully synthetic methods, which became a scaffold for multiple C6 sidechain modifications, further discussed ahead in the context of semi-synthesis.

## 3. Towards the Golden Era: Waksman Platform

Impelled by the remarkable successes at the beginning of the 20th century, Selman Waksman adventured into the realm of drug discovery. In 1937, noticing that complex soil bacteria—actinomycetes—inhibited the growth of other bacteria, Waksman acknowledged that these biological mechanisms, which evolved from competitive growth [44], could become the conceptual basis of a screening platform for antibiotic-producing organisms [45]. From 1939 onwards, it is estimated that his systematic agar overlay process, referred to as the Waksman platform, screened well over 10,000 strains of different microbes [46], which exemplifies the scalability of this method—a key characteristic for the coming successes. Equally important, over 90% of clinical antibiotics derive from actinomycetes [10], making these microbes an antibiotic gold mine of sorts.

The Waksman platform promptly revealed new antimicrobials: actinomycin, streptothricin, fumigacin and clavacin; but it was not until 1944 that a *Streptomyces griseus* strain was found to produce a non-toxic aminoglycoside antibiotic, named streptomycin, which inhibits protein synthesis by binding to the bacterial 30S ribosomal subunit. At the time, it was not possible to patent natural products in the USA, but together with Merck lawyers, Waksman convinced the authorities that purified antibiotics were sufficiently distinct, sparking a new range of business opportunities, a significant stride towards economic stimulus that bolstered the antibiotic golden age. Merck obtained FDA approval for streptomycin [44] and began its commercialization by 1946 for the treatment of tuberculosis and tuberculous meningitis, and later for pathogens outside penicillin’s spectrum of activity [47]. The Waksman platform revealed various antibiotic classes, many of which are the major antibiotic classes currently in clinical use, as described next. 

Chloramphenicol was originally isolated in 1947 from the actinomycete *Streptomyces venezuelae*, thereby introducing the amphenicol class. Chloramphenicol’s antimicrobial activity derives from its reversible binding to the 50S ribosomal subunit, thereby inhibiting bacterial protein synthesis. It was the first FDA-approved broad-spectrum antibiotic, displaying excellent tissue and fluid permeability. However, in the 1960s, various toxicity issues impaired its administration, and it is currently rarely prescribed [48]. Chlorotetracycline marked the introduction of the tetracycline antibiotic class in 1948, which also disrupts protein synthesis by acting on the 30S subunit of the ribosome. Chlorotetracycline, a product of *Streptomyces aureofaciens*, is characterized by its instability at both ends of the pH scale that hampers its bioavailability [49]. 

Macrolides are the second most prescribed class of therapeutic antibiotics, introduced in 1949 with erythromycin and produced by *Saccharopolyspora erythrea*. Erythromycin binds to a 50S bacterial ribosomal target, but its therapeutic use was characterized by instability under acidic conditions and overall poor oral bioavailability [50]. Virginiamycin was the first identified streptogramin, originally isolated from *Streptomyces virginiae* in 1952. Streptogramins are a class of antibiotics formed by two chemically unrelated substances, a polyunsaturated macrolactone and a cyclic hexadepsipeptide. Either group binds to the 50S subunit of bacterial ribosomes, presenting mediocre activity, but their synergistic effect empowers its therapeutic application [51].

Unlike the antibiotic classes described thus far, which target bacterial protein synthesis, glycopeptides disrupt cell wall synthesis. The first antibiotic of the glycopeptide class, vancomycin, was discovered in 1956 to be produced by *Amycolatopsis orientalis* and is currently a last resort antibiotic. Vancomycin interferes with the transpeptidation and transglycosylation steps of the cell wall synthesis, thereby inhibiting cross-linking and cell wall maturation [52]. Similarly, ansamycins differ from protein synthesis inhibitors (e.g., amphenicols, tetracyclines, macrolides and streptogramins) and cell wall synthesis inhibitors (e.g., glycopeptides). For instance, rifamycins inhibit the DNA-dependent RNA polymerase of prokaryotes. Rifamycin B was first isolated in 1959 from *Streptomyces mediterranei* (later classified as *Amycolatopsis mediterranei*), and despite considerably low antimicrobial effect, it introduced a unique metabolic target in bacteria [53]. The discovery of fosfomycin came from the isolation of three *Streptomyces* strains in 1969. Its antimicrobial effect derives from the inhibition of the initial steps of the cell wall biosynthesis pathway, disrupting the action of phosphoenolpyruvate synthetase. However, fosfomycin presents a broad spectrum of activity, making it an appealing antimicrobial [54]. 

Although the first report of a lipopeptide antibiotic dates to 1947 with the discovery of polymyxin E, produced by *Paenibacillus polymyxa*, the therapeutic use of this class was limited to experimentations for a mere couple of years, given multiple concerning adverse effects, but has recently been reconsidered [55]. The production of daptomycin by *Streptomyces roseosporus* was revealed in 1980 and although Eli Lilly and Co. attempted its commercialization, clinical trials were discontinued under the belief that there was a small window between therapeutic efficacy and safety. As such, this calcium-dependent cyclic lipopeptide is seen as the precursor of the lipopeptide class of antibiotics, with surpassing antimicrobial activity in comparison with polymyxin E, albeit limited to Gram-positive pathogens. Interestingly, daptomycin was revived by Cubist Pharmaceuticals and, with dosing adjustments, reached the market by 2003 [56]. Moreover, daptomycin’s mode of action is still unclear: permeabilization and depolarization of the cell membrane being the most probable; interference in cell wall synthesis; and/or disruption of cellular division are other suggestions. Although more cyclic lipopeptides have been described, daptomycin remains the only approved therapeutic antibiotic of this class [57].

## 4. Onto the Medicinal Chemistry Era: Semi-Synthesis

Antibacterial semi-synthesis is the modification of existing scaffolds, or molecular backbones, obtained by a fermentative procedure. Historically, most scaffolds originated from the Waksman platform. Thus, they are the evolutionary outcome of selective pressures, e.g., from the actinomycete-bacteria ‘fight’, and are therefore extremely well fit to reach and bind to their target. However, this does not translate to therapeutic effectiveness or safety, which can often be improved by means of semi-synthesis, alongside its chemical stability, reduction of undesirable side effects, among other features that are crucial in marketing antibiotics, for instance patenting derivatives, which increases profitability of antibiotic development programs, essential for this generally unattractive business. Semi-synthesis began with the catalytic hydrogenation of streptomycin, which resulted in dihydrostreptomycin by 1946, and was characterized by greater chemical stability along with similar antimicrobial activity. Although both streptomycin and its novel derivative quickly made their way to clinical use, eventually their prescription has been reevaluated due to ototoxicity concerns [58]. 

Conversely, it took over a decade before a bioproduction method made penicillin a therapeutic possibility. While the identification of penicillin’s antimicrobial effect preceded its ‘discovery’, it was Fleming’s will power that pushed penicillin beyond only being obtainable in small and unstable quantities. This in turn enabled its semi-synthesis, which expanded penicillin from a single drug to a range of semi-synthetic derivatives constituting an entire class of antibacterial drugs, the beta-lactams. These comprise over 60% of antibiotics for human use [59], with a multitude of subclasses and marketed antibiotics within, as seen in Table 1. The rate at which derivatives with improved properties can be synthetized kept the upper hand against infectious diseases, a key characteristic of semi-synthesis. Nonetheless, resistance to these semi-synthetic antimicrobials has been rapidly increasing, which is thought to be related to their high rate of prescription and highlights the importance of continuously developing novel semi-synthetic derivatives [60]. 

**Figure 2:**
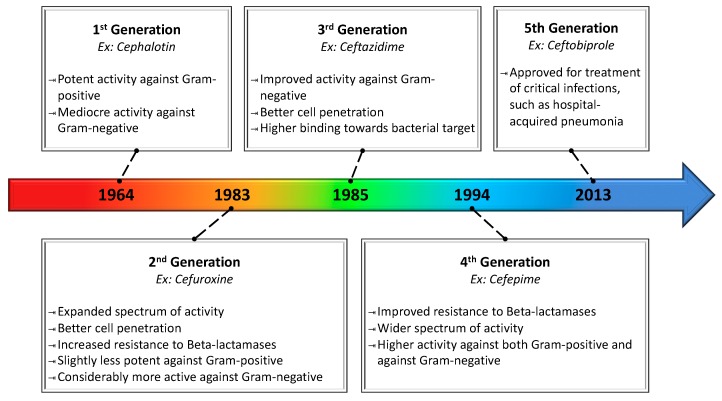
Evolution of cephalosporin characteristics over semi-synthetic generations. Because each generation is the result of adding different molecular groups to 7-ACA, characteristics are not necessarily inherited by succeeding generations. For instance, second-generation cephalosporins had reduced potency against Gram-positive pathogens, despite their otherwise improved properties.

Semi-synthetic penicillins are obtained by producing penicillin G, which is hydrolyzed into 6-APA, purified and later chemically altered, e.g., at the acyl side chain, to achieve various semi-synthetic penicillins [61]. Another beta-lactam example parallel to penicillins is the semi-synthesis of cephalosporins, which have reduced the incidence of both side effects and resistance, alongside an additional site for chemical modification [62]. Cephalosporin C was firstly identified as a metabolite of *Cephalosporium acremonium* in 1948. By 1959, 7-aminocephalosporanic acid (7-ACA) was obtained from its hydrolysis under acidic conditions, thereby introducing the precursor to a multitude of semi-synthetic cephalosporins [63]. Figure 2 exemplifies the evolution of semi-synthetic cephalosporins, their timeline of introduction and the pros and cons of the succeeding generations marketed so far.

Another key illustration of semi-synthesis comes from the catalytic hydrogenolysis of chlorotetracycline (discovered in 1948), which resulted in the semi-synthesis of tetracycline by 1953, although it was later also found to be a natural product [64]. While semi-synthetic cephalosporins are mostly derivatives of 7-ACA, obtained via the addition of different molecular groups at the pair of modifiable sites, i.e., C7 and C3’, semi-synthetic tetracyclines and macrolides are the result of serial structural modifications. Each iteration requires the chemical manipulation of the previous semi-synthetic derivative, which may preserve its advantages, but the number of chemical modifications grows proportionally and become increasingly challenging across a series of semi-synthetic generations. Therefore, less than 10 semi-synthetic tetracyclines were marketed in the last 60 years, in contrast with over 50 commercialized beta-lactam derivatives. However, recent advances in fully synthetic routes have reignited the potential of tetracycline derivative synthesis [49,65], which is crucial given semi-synthesis is one of the major strategies for antibiotic discovery and particularly important in outpacing the evolution of resistance mechanisms.

## 5. From the Ground Up: Fully Synthetic Antibiotics

Fully synthetic antibiotics, beyond introducing novel molecules, enable production at a scale suitable for clinical application. For instance, chloramphenicol became the first fully synthetic antibiotic, whose scaffold originated from a natural product, to reach the clinic in 1949. Unsurprisingly, the rational of semi-synthesis, that of chemically manipulating a scaffold, applies to a fully synthetic antibiotic like chloramphenicol. In fact, replacing the nitro group with methanesulfonyl resulted in thiamphenicol in 1952, which overcame the most concerning toxicity issues and had greater antimicrobial effect, thereby improving its clinical application [66]. The discovery in 1953 of the natural product azomycin found little clinical application but introduced the nitroimidazole class. In 1962, the search for optimized derivatives revealed metronidazole, currently produced with a fully synthetic protocol, which is active against the trichomoniasis parasite. Curiously, its activity against anaerobic bacteria was a fortuitous discovery, for which it is still in use [67]. Analogously to metronidazole, the natural product fosfomycin only had reasonable clinical application once a racemic synthesis protocol was developed by Merck, and is still prescribed today [54]. While the advantages of chemically synthetizing natural products are straightforward, fully synthetic antibiotics also resulted in novel scaffolds. Given synthetic analogs of pyrimidine and purine bases inhibit bacterial growth, a diaminopyrimidine derivative named trimethoprim was introduced in 1962 [68], but only commercialized in 1969 together with sulfamethoxazole due to in vitro synergies, which are being questioned in light of recent in vivo observations [69]. 

Most of the fully synthetic antibiotics discussed have limited application to uncomplicated infections or as an economic alternative in developing countries. The quinolone class, which was unexpectedly discovered as a by-product of the synthesis of the antimalarial compound chloroquine, despite limited activity, was an important scaffold in the synthesis of nalidixic acid in 1962 [70]. Three more generations, the fluoroquinolones, were later obtained via chemical modification. Quinolones are currently the third most prescribed antibiotic to outpatients, behind macrolides and beta-lactams [71], and their antimicrobial effect is traced to the formation of a DNA gyrase-quinolone-DNA complex, which hampers replication and induces cellular death in both Gram-positive and Gram-negative pathogens. Another major antibiotic class, macrolides, are produced by semi-synthesis from erythromycin, which may involve simpler routes (e.g., four steps to derive azithromycin) up to more intricate modifications (e.g., 16 steps for the drug candidate solithromycin). The recent report of a fully synthetic protocol that yielded over 300 macrolides [72] brings new hope to this class of antibiotics and portrays the importance of the fully synthetic platform up to this day, not only in facilitating the synthesis, but also increasing the diversity of the antibiotics available.

The case of fully synthetic beta-lactams is of paramount relevance since it allowed to more intricate antibiotics to be synthetized, eventually leading to a panoply of subclasses. Two important examples are the subclasses of carbapenems and monobactams. Carbapenems have a similar core structure to penicillins, differing at the C2–C3 double bond and the replacement of C1 sulfur for carbon, yielding improved potency, spectrum of activity, and better resistance to the action of beta-lactamases. Currently, 10 carbapenems derivatives have been marketed, or are under clinical development, since their discovery in 1985. Given that carbapenems have the widest activity spectrum among beta-lactams, including resistant pathogens, they are currently a first in line option for treating multidrug-resistant infections [73]. Likewise, monobactams have higher stability regarding beta-lactamases and are a promising way forward. These monocyclic beta-lactams were introduced to the clinic in 1984 with aztreonam and are currently being developed towards siderophore moiety, a Trojan horse strategy that uses the bacterial iron uptake machinery to facilitate entry into Gram-negative bacteria [74].

The class of oxazolidinones is divided into two groups differing in their mode of action. The first acts on cell wall biosynthesis and was introduced with the natural product cycloserine in 1952, which is currently produced by synthetic means. Cycloserine is still used as a second-line therapeutic option for tuberculosis, especially in its multidrug-resistant form. The other group of oxazolidinones was found in 1984 to target protein synthesis and, despite reasonable antimicrobial activity, presented limiting toxicity issues [75]. From these, the DuPont group synthetized various derivatives from which resulted the discovery of linezolid, approved in 2000 as the first novel antibiotic class since the discovery of nalidixic acid, with almost half a century discrepancy [76]. Although no major resistance to linezolid has been reported, its limited effectiveness against Gram-positive bacteria and toxicity in prolonged treatments limits its therapeutic use as a last resort alternative against complicated cases of multidrug-resistant pathogens. Over the last decade, there has been substantial interest in developing novel oxazolidinones, given its low resistance profile, thus a handful of companies have been developing novel analogues [77]. Semi-synthesis, along with complete chemical routes, have catalyzed the dawn of the medicinal chemistry era, which together with the Waksman platform, have yielded the vast majority of clinically relevant antibiotics, characterized by increasing potency and diminishing side effects with succeeding iterations, which gave mankind the upper hand on infectious diseases. 

## 6. Advent of Genomics: Target-Based Screening

**Figure 3:**
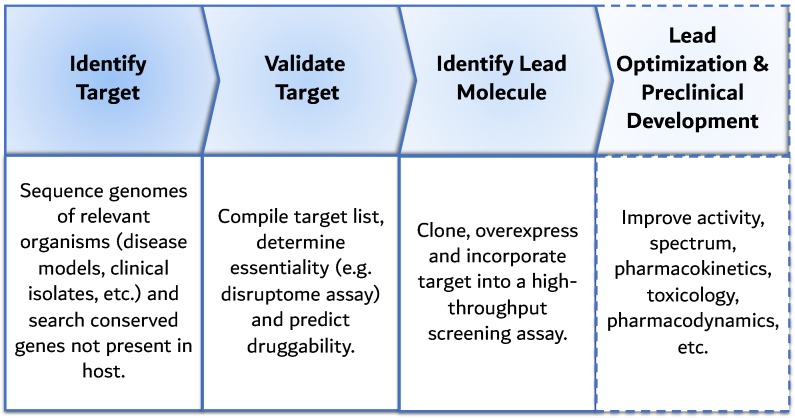
Schematic representation of the target-based antibiotic discovery platform: potential targets are identified from the genome sequence of pathogens and the host, the products of genes exclusive and essential for bacteria are incorporated into high-throughput screening assays, which identify drug candidates suitable for lead optimization and preclinical development. The latter falls outside the scope of antibiotic discovery, thus it is not discussed.

After the successes of the antibiotic golden age, the discovery rate of the underlying ADPs has decreased, along with an increase in class and multidrug-resistance mechanisms, which has weakened the therapeutic efficacy of the antibiotic arsenal and revived the issue of infectious diseases. The need for a new strategy coincided with the genomics era, which redefined the scientific paradigm governing antibiotic discovery and shaped new high-tech platforms. During the genomics era (1995–2004), the total number of sequenced microbial genomes increased from 3 to over 200 [78], and in the post-genomics era (2004–2014) reached a staggering 30,000 [79]. In this context, the first platform to arise was based on comparative genomics, where novel targets essential for pathogen survival were identified from repositories of sequenced and annotated genomes. These targets can encode pathogenicity mechanisms, highlighted by comparing genome sequences of pathogenic and non-pathogenic strains. Furthermore, comparing these genomes to those of the host rejects targets that are not exclusive to the pathogen, which minimizes drug–host interactions, resulting in fewer therapeutic side effects. Figure 3 resumes the target-based ADP: after target discovery, target validation follows by evaluating if they are essential for bacterial survival, e.g., with knockout analysis and/or mutational studies. After, the target is cloned, overexpressed and incorporated in a high-throughput screening (HTS) assay to search for binding agents from chemical libraries.

Given that a manageable number of proteins are exclusive and conserved in bacteria, new MOAs were expected to surface, so some companies launched pioneering target-based screening programs. GlaxoSmithKline developed a target list of over 300 bacterial genes from 1995 to 2001, of which approximately 160 were considered essential for survival, and deemed ‘druggable’ in the search for broad-spectrum antibiotics [80]. Elitra pharmaceuticals, one of the top 10 start-up companies of 2001, submitted patents on over 4000 targets after developing a proprietary strategy that identified essential genes in several pathogens [81]. Although target-based screening is suitable for finding potent inhibitors of said targets, their inability to reach their target, due to the low permeability of bacterial membranes or the action of efflux pumps, hinders their activity in vivo. In a physiological context, the bacterial cell wall is a very efficient barrier against most small molecule drugs. Moreover, said targets may present functional redundancy. Alongside the aforementioned difficulties, the target-based screening approach also failed because not all targets could be readily cloned, purified and incorporated into in vitro screening assays; and in some cases, the oversimplified environment of the assay excludes cofactors and lacks sensitivity for off-target effects. For instance, Merck found that low guanine–cytosine Gram-positive pathogens have increased resistance to fatty acid biosynthesis targets when grown on media mimicking the human host [82], which a target-based assay cannot consider. Also, single gene targets are prone to single point mutations conferring resistance, thus are more likely to select resistant mutants.

Despite the massive bacterial genome sequencing coupled with the development of bioinformatics tools to analyze said sequences, there are still many genes whose biological function has not been characterized. Moreover, genetic diversity further complicates target-based screening at the level of model organism selection, e.g., GlaxoSmithKline researchers reported an unrelated copy of genes conferring resistance in 20% of clinical isolates [83]. Ultimately, antibiotic discovery remains a challenging affair unattainable with an exclusively target-based genomics approach, and many consider the comparative genomics platform as rather unsuccessful, since not a single new drug was discovered [84]. Nonetheless, it sparked a quest towards understanding bacterial physiology, which had unquestionable positive implications in the development of antimicrobial chemotherapy. The reductionist approach of target-based screening, e.g., analyzing a single gene/protein (target) outside its biological context, evolved towards a more holistic phenotypic and pathway-based analysis. Subsequent platforms stemmed from taking a step back and reviving whole-cell screening, which was the basis of the Waksman platform, and bears the intrinsic advantage that lead compounds can interact anywhere on the pathway, on multiple constituents of the network or even on different metabolisms, and most importantly, replicating in vivo conditions.

## 7. Reverse Genomics: Revival of Cell-Based Screening

**Figure 4:**
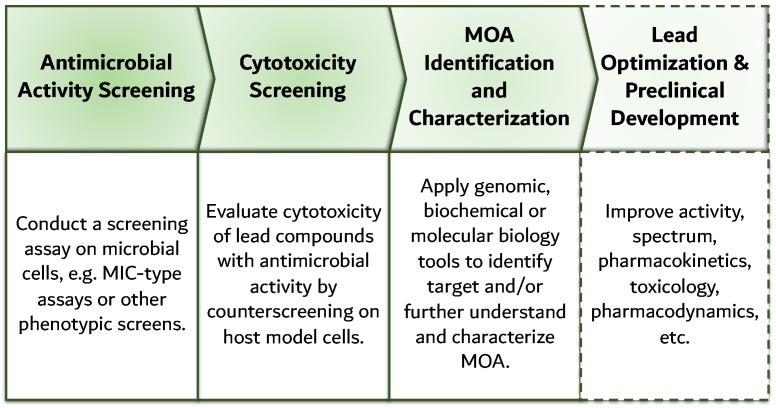
Schematic representation of the cell-based antibiotic discovery platform: drug candidates are identified from cell-based screening assays, a counter-screen excludes cytotoxic compounds, and subsequently genomics tools are applied to identify MOA. Although MOA is not a requisite, it may facilitate lead optimization and preclinical development, for instance, structural information on the target can enable a rational modification of the drug candidate.

The case of anti-tuberculosis drug discovery is a good example of this change in strategy: researchers moved away from target-based ADPs and returned to cell-based screening [85], with greater success in the discovery of novel, more diverse, lead molecules for subsequent optimization [86]. In general, cell-based screening results in higher variability and more complex data than the binary hit/no-hit of target-based screening, which is more difficult to relate with biologic phenomena. In cell-based assays, after a positive hit, e.g., an interaction of a drug with a microorganism such that its phenotype becomes altered, counter-screening with human cells allows for cytotoxic evaluation of drug candidates with antimicrobial activity. Cell-based ADPs first identify antimicrobial activity and only later endeavor to characterize MOA, and thus are also named reversed genomics, as represented in Figure 4. This is not necessarily a limitation as the FDA does not require the identification of the molecular target to initiate clinical trials, or to obtain marketing approval [87]. 

In a broad sense, cell-based assays include screening large libraries in a systems-based mentality in order to evaluate the complex network of responses that antibiotics elicit [88], and are often termed phenotypic screening. Typically, if said screening probes phenotypic changes free of target hypothesis, the term target-agnostic may be applied. Moreover, cell-based screening may follow a chemocentric approach, e.g., on compounds and its derivatives presenting a known biological effect. The development of cell-based screening methods has been of paramount importance and in its simpler form, these are centered on determining the Minimum Inhibitory concentration (MIC) to quantify antimicrobial activity. MIC assays are still relevant given that they complement other ADPs, for instance, Seiple et al. [72] developed a fully synthetic protocol for producing macrolide derivatives, where MIC assays were used to evaluate antimicrobial activity. Efforts to extend cell-based screens beyond conventional MIC assays, which do not provide insight on the MOA of candidate molecules, have been directed to developing assays that measure either: mitochondrial activity, by measuring a fluorescent product of a mitochondrial reaction; cellular integrity, evaluating the release of intracellular enzymes or the uptake of dyes that are impermeable when the cell is healthy; or measuring ATP content, etc. The reporter gene technology is still predominant, where the activation and expression of a gene, which yields a quantifiable signal, e.g., luminescence or fluorescence, ‘reports’ biomolecular interactions. For instance, Hutter et al. [89,90] developed a HTS assay with a panel of twelve *Bacillus subtilis* strains, modified with luciferase reporter genes, to indicate the MOA of various antibiotics with sensitivity ranging from generic pathways, antibiotic class and the specific MOA of some drugs.

Alternatives to MIC-type assays require genetic manipulation and/or the use of a label, either in the form of a fluorescent or radioactive molecule, or a reporter gene. This is a limiting factor since, on the one hand, genetic manipulation implies a priori knowledge and on the other hand, the indication of gene transcription using a reporter gene may not always be coherent with alterations of enzymatic activity, thereby crippling the inherent sensitivity of these assays. Moreover, these signal transduction events can take considerable time to become detectable, thus limiting assay capacity and throughput [91]. In addition to the impact of a reporter gene, some of these labeled assays are limited on miniaturization. Despite said issues, cell-based screening still contributes greatly towards advancing antibiotic discovery, for instance, in neglected diseases such as malaria and human African trypanosomiasis [92]. For the latter, phenotypic screening lead to the discovery of fexinidazole (a nitroimidazole) which has been recently approved as the first oral therapy for human African trypanosomiasis and Chagas disease [93].

Eder et al. reviewed the discovery platforms of first-in-class small molecule drugs, in particular the role of target- versus cell-based screening [94]. First-in-class drugs act on a new target or biological pathway. Between 1999 to 2008, phenotypic screening was more productive. However, in the period up to 2013, target-based approaches delivered most first-in-class drugs. Given the period ranging from the burdensome process of drug discovery until commercialization, there is a latency between the timeline of said review and the timeline presented in this manuscript. Concerning antibiotics, from 2000 to 2015, only five first-in-class new drugs were marketed: linezolid, daptomycin, retapamulin, fidaxomicin and bedaquiline [95]. Retapamulin binds to the 50S ribosomal subunit, fidaxomicin acts at the “switch” region of the bacteria RNA polymerase and bedaquiline specifically inhibits the ATPase of *Mycobacterium tuberculosis*. From these five new drugs, three are derived from natural products (daptomycin, retapamulin and fidaxomicin), and only two were chemically synthesized (linezolid and bedaquiline) [90]. 

Historically, the success of antibiotic therapy relied on the discovery of natural scaffolds that were chemically optimized or produced. As such, it remains a rational decision to continuously develop screening strategies that probe natures repositories [96], especially using cell-based assays [97]. In fact, the major antibiotic scaffolds currently in use are derived from natural products, except for fluoroquinolones, sulphonamides and trimethoprim [98]. New cell-based ADPs should identify molecules with antimicrobial activity without the limitations of a label. Moreover, these assays would ideally provide insight on MOA whilst being capable of screening very large libraries, as isolated projects have very low rates of success [99]. 

## 8. Post-Genomics: Transcriptomics, Proteomics and Lipidomics

Biological research tends towards specialization, through increasingly focused and localized research; however, system-wide understanding of the biological constituents and their interactions is gaining importance. It is now possible to extract, handle and interpret information from much higher dimension and diverse origins, such as transcripts (i.e., transcriptomics), proteins (proteomics), and other molecules such as lipids (lipidomics), etc. The influence of these omics technologies on the field of antibiotic discovery is undisputable, especially in understanding antibiotics MOA, identifying novel targets, and supplying insights to bacterial metabolism and physiology. Given the importance of screening in the ADPs discussed thus far, this mindset should be the backbone of future platforms. However, neither transcriptomics, proteomics nor lipidomics have matured to the throughput capacity of cell-based screening assays, and therefore are not the core technology of any ADP. These technologies convey insight on the biomolecules they probe, and not the holistic dynamics of bacterial metabolism, thereby serving as complementary, albeit crucial, tools for the antibiotic discovery process. Since a post-genomics ADP is yet to be developed, this section focusses on the technological basis and contribution of these post-genomics techniques to the field of antibiotic discovery.

Initial transcriptomics technologies were hybridization-based, e.g., Northern Blotting and microarrays [100]. Microarrays became the reference by mid-1990s [101] until next-generation sequencing extended to transcriptomics, which copes better with high genetic variation and non-specific hybridizations, as well as being label-free, e.g., unbiased and with a greater upper limit of detection. RNA-seq outperforms microarrays (90% versus 76%) in predicting differentially expressed genes. However, both technologies similarly estimated the MOA of anti-cancer drugs [102]. However, RNA-seq enables studying non-coding RNA, which has a regulatory role in microbial responses to antibiotics, and therefore can be an alternative for new antimicrobial targets and/or novel combinatorial therapies [103]. Although next-generation sequencing [104], unbiased transcriptomics [105] and non-coding RNA [106] technologies have been applied to drug discovery in general, few studies discuss their application to antibiotic discovery. Whole-genome expression profiling elucidates the molecular and cellular responses to antibiotic stresses, which is particularly helpful for MOA determination, still a major gap in the field of antibiotic discovery. For instance, Salvarsan’s MOA remained unclear over a century since its discovery, and its chemical structure has only recently been fully elucidated [107]. In general, antimicrobials of the same class, thus with similar MOA, give rise to analogous transcriptional responses, which provides insight on the MOA of uncharacterized antibiotics [108]. For instance, the cell-based HTS assay developed by Hutter et al. [89,90] used transcriptomics to characterize the effect of various antibiotics, which then guided the genetic manipulation of a bacterial panel that ‘reports’ lead molecules MOA. Additionally, these signature responses are also being used to elucidate resistance mechanisms [109].

Genome expression technologies expand beyond the transcript level to biological events at the level of proteins. Not only do these occur without transcriptome alterations, but the instability of bacterial RNA raises both conceptual and technological limitations, which stress the need to complement transcriptomics with proteomics. Early proteomics studies relied on 2D gel-based assays or on Difference Gel Electrophoresis [110], which require high-purity protein samples given their little sensitivity for low-abundance proteins, co-migration of proteins, and different modifications on the same protein [111]. Moreover, gel-based techniques are laborious, poorly automatable, and therefore difficult to apply in large-scale studies, so the evolution of MS coupled with chromatographic separation presents an alternative [112]. Proteomics has contributed towards identifying novel antimicrobial targets [113], understanding resistance mechanisms to therapeutic antibiotics [114], and to elucidate MOA [115], although unable to fully characterize MOA [116]. Importantly, the application of transcriptomics and proteomics technologies sheds new light on the function of various genes, leading to updates on existing annotations, and to improved understanding of bacterial metabolism and physiology. Although not at the core of any ADP, these technologies complement other ADPs, revealing information that is building the way forward. Since proteins interact with different biomolecules, including nucleic acids and lipids, specialized techniques have been developed to probe said interactions [117]. Moreover, within the realm of proteomics, the field of phosphoproteomics has ‘spun-off’. Although this type of post-translational modifications was thought to be exclusive to eukaryotes, it affects bacterial homeostasis, virulence [118], and signal transduction [119]. Virulence mechanisms are interesting since the machinery used by bacteria to cause disease, for instance tyrosine kinases and phosphatases, are structurally different from the hosts and therefore can be exclusively targeted. Further descriptions on phosphoproteomics for drug discovery exist, albeit outside the scope of infectious diseases [120].

Understanding the physiological role of lipids, especially at the molecular level, has been considerably limited due to a technological gap that is being filling with very selective and sensitive lipidome characterization studies using MS, and combining various targeted and non-targeted approaches [121]. To achieve the required lipid separation, various chromatographic methods are routinely applied in combination with MS, for instance hydrophilic interaction liquid chromatography or gas chromatography [122]. Besides their structural function, lipids take part in a panoply of different biological events including signaling, trafficking and even metabolite functions. Regarding infectious diseases, an example of the application of lipidomics is the characterization of pathogenic microbe’s cell wall, thereby unveiling its regulation and role in pathogenesis. This has revealed essential enzymes involved in fatty acid synthesis that are conserved across many of the most clinically relevant pathogens, e.g., FabI, FabH, FabF and acetyl-CoA carboxylase. As such, inhibitors of said enzymes are promising targets for future development [123], especially for persistent mycobacteria infections that use fatty acids as a carbon source [124]. 

## 9. Post-Genomics: From Metabolomics towards Meta-omics

Gene expression data from transcriptomics and proteomics faces challenges, for instance, increases in RNA levels might not coherently result in changes at the protein level, added to conceptual and technological limitations associated with bacterial RNA instability, and differences in protein levels are often poor estimators of metabolic activity. Consequently, interest in small-molecule metabolites has also emerged [125]. Metabolomics provides a more in-depth view of the biological reality governing microbial metabolism, using complex analytical methods like NMR and chromatographic techniques associated with MS, alongside advanced data analysis algorithms [126,127]. Since bacterial responses to antibiotics begins rapidly and encompasses a variety of pathways, metabolomics is well suited to elucidating the MOA. For instance, Hoerr et al. [128] explored NMR-based metabolomics to differentiate the MOA of nine antibiotics on *Escherichia coli*. Moreover, metabolomics complements other omics, for instance, rhodomyrtone’s antimicrobial activity was identified via phenotypic screening, but its MOA was revealed with proteomics and metabolomics. Specifically, rhodomyrtone cripples capsule biosynthesis enzymes and metabolites of *Streptococcus pneumoniae* [129]. Additionally, it is possible to construct metabolic networks that aggregate catalytic activity (i.e., enzymes) alongside its coding and expression (i.e., genes, and their transcriptional and translational control). Over 50 networks of different organisms have been described, which sparked a new approach for antimicrobial target discovery [130]. 

Metagenomics, and meta-omics in general, have reinforced natural product discovery, which has had a central role in antibiotic discovery, and chemotherapy in general, ranging from oncological to immunologic treatments, e.g., approximately 50% of all FDA-approved therapeutics are natural products or their derivatives [131]. Metagenomic studies estimated that only 10% of natural products have been identified, so the suggestion that only 1% of the complete natural products repository has been investigated comes as no surprise [132]. Therefore, the search for new drugs from natural sources is being pursued with renewed hopes [133]. In this regard, sampling new natural product sources, such as plants and marine organisms [134], and endophytes or epiphytes [135], is expected to reveal an even wider range of metabolic pathways with potential therapeutic applications. Moreover, exploring microorganisms unculturable in traditional laboratory conditions, or certain pathways not activated in typical laboratory conditions, requires efforts to develop adequate protocols. Given the meta-omics revelation of natures ‘untapped’ repositories, these could very well be the next ‘gold mine’ after the actinomycete-fueled Waksman platform, thereby justifying such efforts.

An interesting device, the iChip, allows for the high-throughput cultivation of microbial species in their natural habitat, with a growth recovery of 50% versus 1% of traditional recovery methods, thereby giving access to otherwise ‘uncultivable’ microorganisms [136]. The iChip was used to collect extracts from 10,000 isolates from which a new species of Beta-proteobacteria, thought to belong to a new genus related to *Aquabacteria*, was shown to produce an antibiotic named teixobactin, a peptidoglycan synthesis inhibitor. Teixobactin is mostly active against Gram-positive pathogens, some of which are drug-resistant, and its bactericidal activity even surpasses that of vancomycin (a last resort antibiotic), along with no indication of resistance mechanisms currently existing [137]. Metagenomics enables a different approach, instead of attempting to grow these ‘uncultivable’ microorganisms, sequences of interest can be identified from the metagenomes, which can then be cloned and expressed in laboratory-friendly microbes. This avoids in situ cultivations, like with the iChip, or the burdensome tasks of deciphering the conditions required for growth or activation of unexplored pathways, and could provide novel molecules for antibiotic discovery [138]. 

The interaction of antibiotics with the human microbiome has also been enabled by meta-omics, which has created further opportunities for antibiotic discovery [139]. In fact, human-associated metagenomic studies revealed gene clusters with antibiotic potential. For instance, in the case of *Staphylococcus lugdunensis* nasal colonization, these commensal bacteria inhibit the presence of *Staphylococcus aureus* strains, thereby preventing opportunistic infections. This effect was traced to the production of lugdunin, a novel class antibiotic (macrocyclic thiazolidine peptides) produced by *S. lugdunensis*, which has bactericidal activity on key pathogens and importantly, presents a reduced risk of resistance development [140]. Likewise, lactocillin, a novel thiopeptide antibiotic, was identified from the vaginal microbiota and demonstrated considerable activity against typical pathogens [141]. On a different note, recurrent *Clostridium difficile* infections have been treated with complete microbiome transplantation [142], which is a ‘brute-force’ alternative in comparison to pinpointing the key molecular agent responsible for the regulation between commensal flora and pathogenic agents. These studies suggest that either introducing healthy microbiota, the targeted manipulation of commensal microbial populations, or even the purified molecular agents of commensal bacteria can be novel therapeutics, which has been enabled by meta-omics technologies. 

As seen, the technologies introduced in the post-genomics era have contributed towards new opportunities in antibiotic research, although these have not been at the core of any ADP. The case of teixobactin, for instance, heavily relied on the revelations brought by meta-genomics and the technologies required to build a device such as the iChip. However, identifying which of the molecules recovered with the iChip have antimicrobial activity, along with insights into their MOA, were revealed with cell-based assays in a reverse genomics platform. Since phenotypic screening has had greater success in revealing first-in-class molecules, it is a great starting point for ADPs. However, the drawback is the reduced mechanistic information derived, for which the omics technologies supply accelerated insight on the MOA, including the molecular target and its regulation. Then, with the required mechanistic information, target-based screening can be applied in order to optimize lead molecules into best-in-class medicines [143]. Although most new antibiotics in late clinical development belong to existing classes [144], the paradigm of combining target- and cell-based screening brings renewed hope moving forward. Additional opportunities may arise from revisiting compounds that were discontinued at early stages of their development. In that sense, Farrell et al. [145] launched AntibioticDB, a database of antibiotics at all stages of development, including those that were discontinued. While some compounds were legitimately abandoned, e.g., in light of clinical results, due to toxicity issues or inferior effectiveness, the majority were discontinued for unknown reasons, and some were discarded for circumstantial reasons. If re-evaluated with novel chemical synthesis methods, or with post-genomics technologies, many abandoned compounds may prove to be effective therapeutics. The case of daptomycin is a good example of how a compound can be revived, nearly 20 years after its abandonment, and still become the most financially successful intravenous antibiotic in the US [56].

## 10. Conclusions

Once considered a resolved health issue, infectious diseases have resurfaced as a topic requiring urgent action. Antibiotic discovery has come a long way since the success of the Waksman platform, semi-synthesis and fully synthetic ADPs. As seen, the establishment of systematic procedures—platforms—was crucial for the discovery of the major antibiotic classes in use. Given the limitations of target-based screening, cell-based ADPs were revived during the genomics era. While some consider the genomics era platforms disappointing, the importance of the lessons learned should not be minimized. Considerable technological advances have given researchers unprecedented access to biological events and repositioned the mind-set for antibiotic research in a systems biology context. Paradoxically, in the field of antibiotic discovery, the more we know the less we can discover. Although not at the core of any ADPs, omics technologies have been proven of unquestionable value as auxiliary tools for antibiotic discovery. Importantly, cell-based screening requires MOA characterization, for which omics technologies are indispensable. Despite offering added-value information on biological events, their reduced throughput capacity alongside complementarity, in terms of resourcing to multiple omics simultaneously, implies a limited application in ADPs aiming to screen large libraries, for instance the reservoir of untapped natural products, which is likely the next antibiotic ‘gold mine’. There is a void between phenotanypic screening (high-throughput) and omics-centered assays (high-information), where some mechanistic and molecular information complements antimicrobial activity, without the laborious and extensive application of various omics assays. Given the novelty of the various omics technologies, we are yet to extract their full potential and it seems feasible that these technologies will mature to fulfill this gap. Alternatively, innovative technologies favoring high-throughput may be developed, even by sacrificing molecular sensitivity to some extent. In any case, the increasing need for antibiotics drives the relentless and continuous research on the foreground of antibiotic discovery. This is likely to expand our knowledge on the biological events underlying infectious diseases and, hopefully, result in better therapeutics that can swing the war on infectious diseases back in our favor. 

## Figures and Tables

**Table 1:** Beta-lactam subclasses highlighting their diversity with examples of marketed antibiotics.

Subclasses	Examples of Marketed Antibiotics
Penicillins	Penicillin G, Penicillin V, Ampicillin, Amoxicillin, Bacampicillin, Cloxacillinm, Floxacillin, Mezlocillin, Nafcillin, Oxacillin, Methicillin ^a^, Dicloxacillin ^a^, Carbenicillin ^b^, Idanyl ^b^, Piperacillin ^b^, Ticarcillin ^b^
Cephalosporins	Cefalothin ^c^, Cephradinea ^c^, Cefadroxyl ^c^, Cefazolin ^c^, Cephalexin ^c^, Cefuroxine ^d^, Cefaclor ^d^, Cefotetam ^d^, Cefmetazole ^d^, Cefonicid ^d^, Cefixime ^e^, Ceftibuten ^e^, Cefizoxime ^e^, Ceftriaxone ^e^, Cefamandol ^e^, Cefoperazone ^e^, Cefotaxime ^e^, Proxetil ^e^, Cefprozil ^e^, Ceftazidime ^e^, Cefuroxime Axetil ^e^, Cefpodexime ^e^, Cefepime ^f^, Ceftobiprole ^g^
Other Minor Subclasses	Flomoxef ^h^, Latamoxef ^h^, Cefoxitin ^i^, Loracarbef ^j^, Imipenem ^j^, Meropenem ^j^, Panipenem ^j^, Aztreonam ^k^, Carumonam ^k^

^a^ Penicillinase-resistant penicillin; ^b^ Anti-pseudomonal penicillin; ^c^ First-generation cephalosporin; ^d^ Second-generation cephalosporin; ^e^ Third-generation cephalosporin; ^f^ Fourth-generation cephalosporin; ^g^ Fifth-generation cephalosporin; ^h^ Oxycepham; ^i^ Cefam; ^j^ Carbapenem; ^k^ Monobactam.

## Supplementary Materials

Supplementary File 1
