# Supplementary material for: Antibiotic Discovery: Where Have We Come from, Where Do We Go?
Source: Antibiotics (Basel). 2019 Apr 24;8(2):45. doi: 10.3390/antibiotics8020045 (PMC6627412; doi:10.3390/antibiotics8020045)
Supplement: Supplementary File 1 [file antibiotics-08-00045-s001.zip › CephalosporinEvolution.pdf]

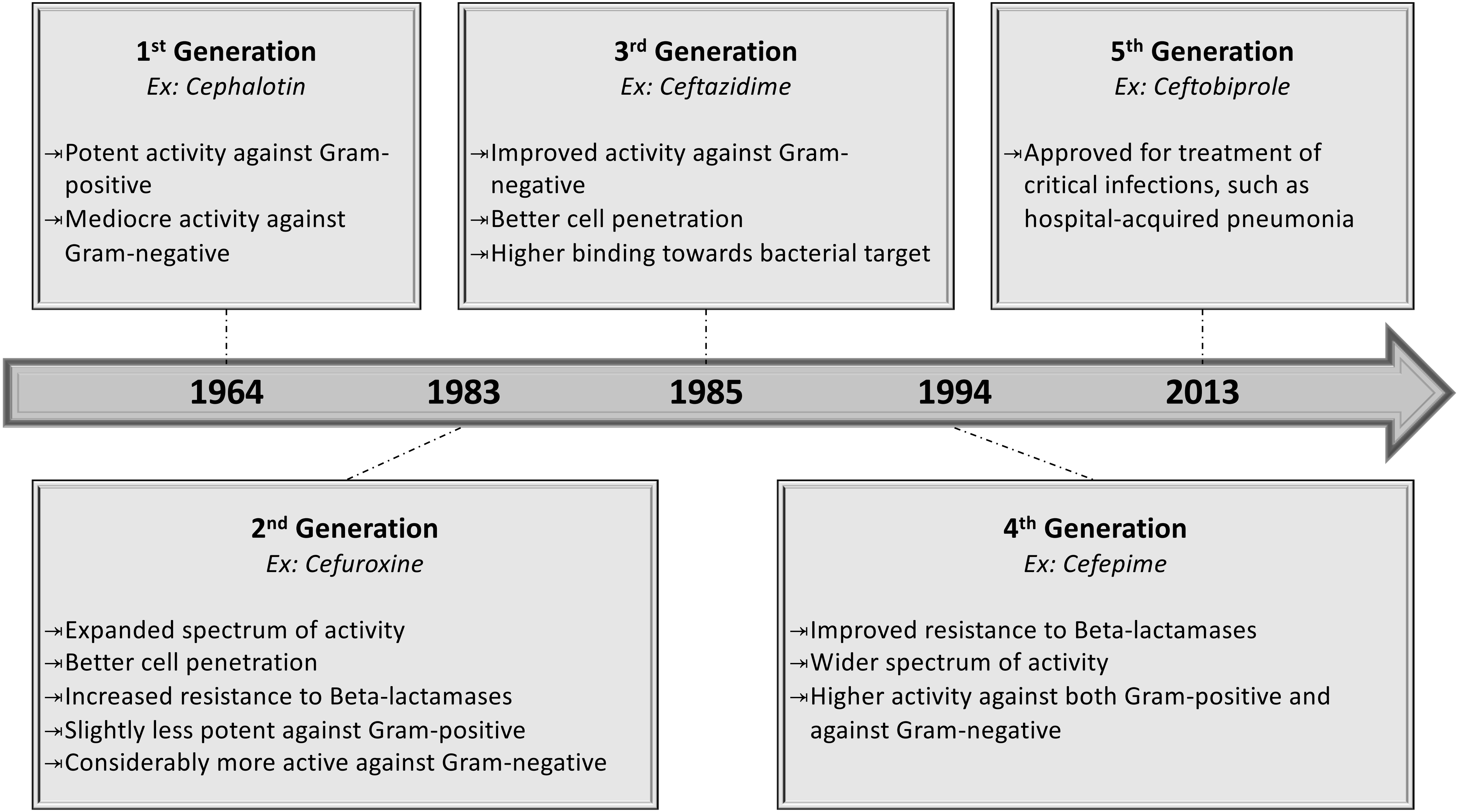
1st Generation
Ex: Cephalotin
→Potent activity against Gram-positive
→Mediocre activity against Gram-negative
1964
1983
2nd Generation
Ex: Cefuroxine
→Expanded spectrum of activity
→Better cell penetration
→Increased resistance to Beta-lactamases
→Slightly less potent against Gram-positive
→Considerably more active against Gram-negative
3rd Generation
Ex: Ceftazidime
→Improved activity against Gram-negative
→Better cell penetration
→Higher binding towards bacterial target
1985
1994
4th Generation
Ex: Cefepime
→Improved resistance to Beta-lactamases
→Wider spectrum of activity
→Higher activity against both Gram-positive and against Gram-negative
5th Generation
Ex: Ceftobiprole
→Approved for treatment of critical infections, such as hospital-acquired pneumonia
2013
