# Supplementary material for: Antibiotic Discovery: Where Have We Come from, Where Do We Go?
Source: Antibiotics (Basel). 2019 Apr 24;8(2):45. doi: 10.3390/antibiotics8020045 (PMC6627412; doi:10.3390/antibiotics8020045)
Supplement: Supplementary File 1 [file antibiotics-08-00045-s001.zip › TargetBasedADP.pdf]

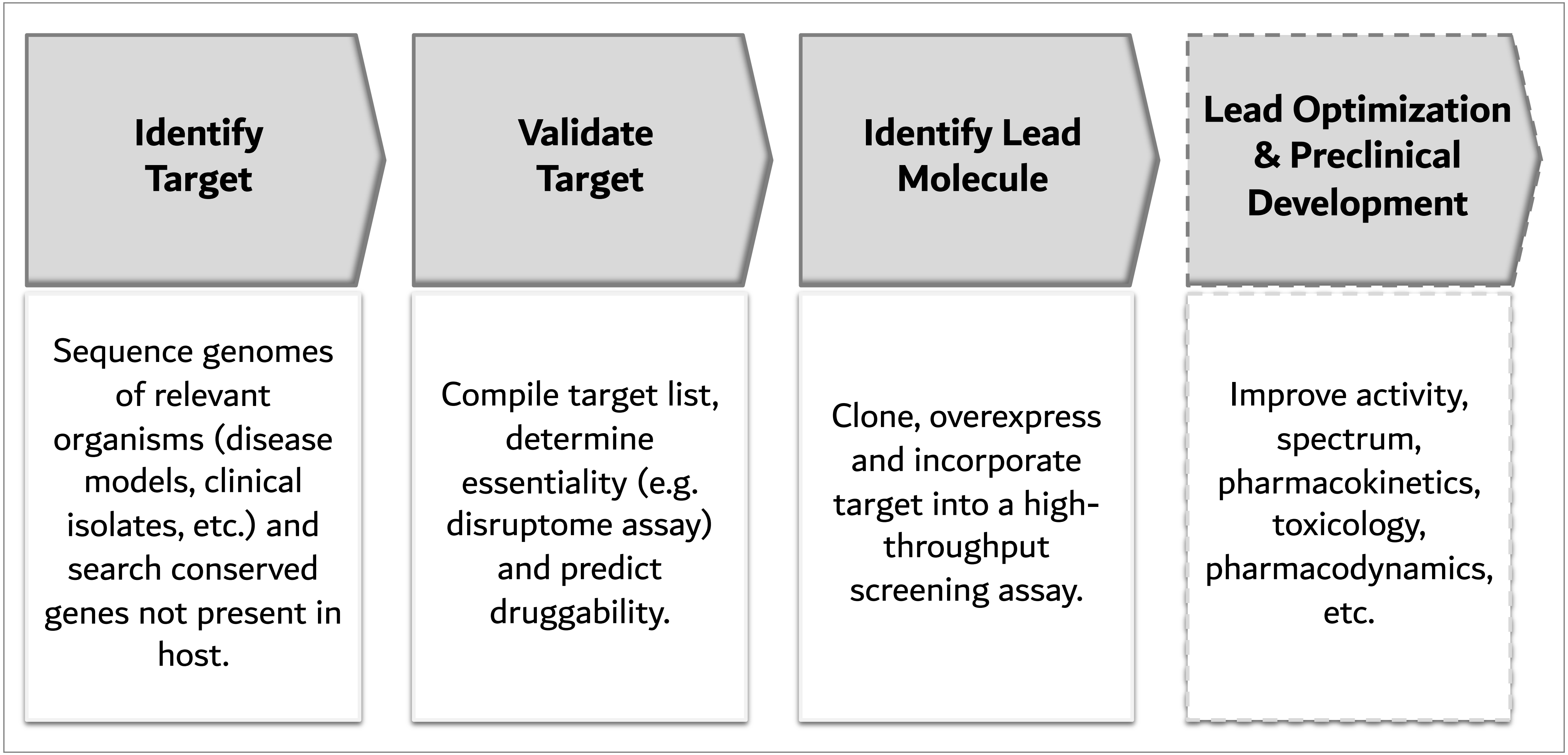
Identify Target
Validate Target
Identify Lead Molecule
Lead Optimization & Preclinical Development
Sequence genomes of relevant organisms (disease models, clinical isolates, etc.) and search conserved genes not present in host.
Compile target list, determine essentiality (e.g. disruptome assay) and predict druggability.
Clone, overexpress and incorporate target into a high-throughput screening assay.
Improve activity, spectrum, pharmacokinetics, toxicology, pharmacodynamics, etc.
