# Supplementary material for: Antibiotic Discovery: Where Have We Come from, Where Do We Go?
Source: Antibiotics (Basel). 2019 Apr 24;8(2):45. doi: 10.3390/antibiotics8020045 (PMC6627412; doi:10.3390/antibiotics8020045)
Supplement: Supplementary File 1 [file antibiotics-08-00045-s001.zip › CellBasedADP.pdf]

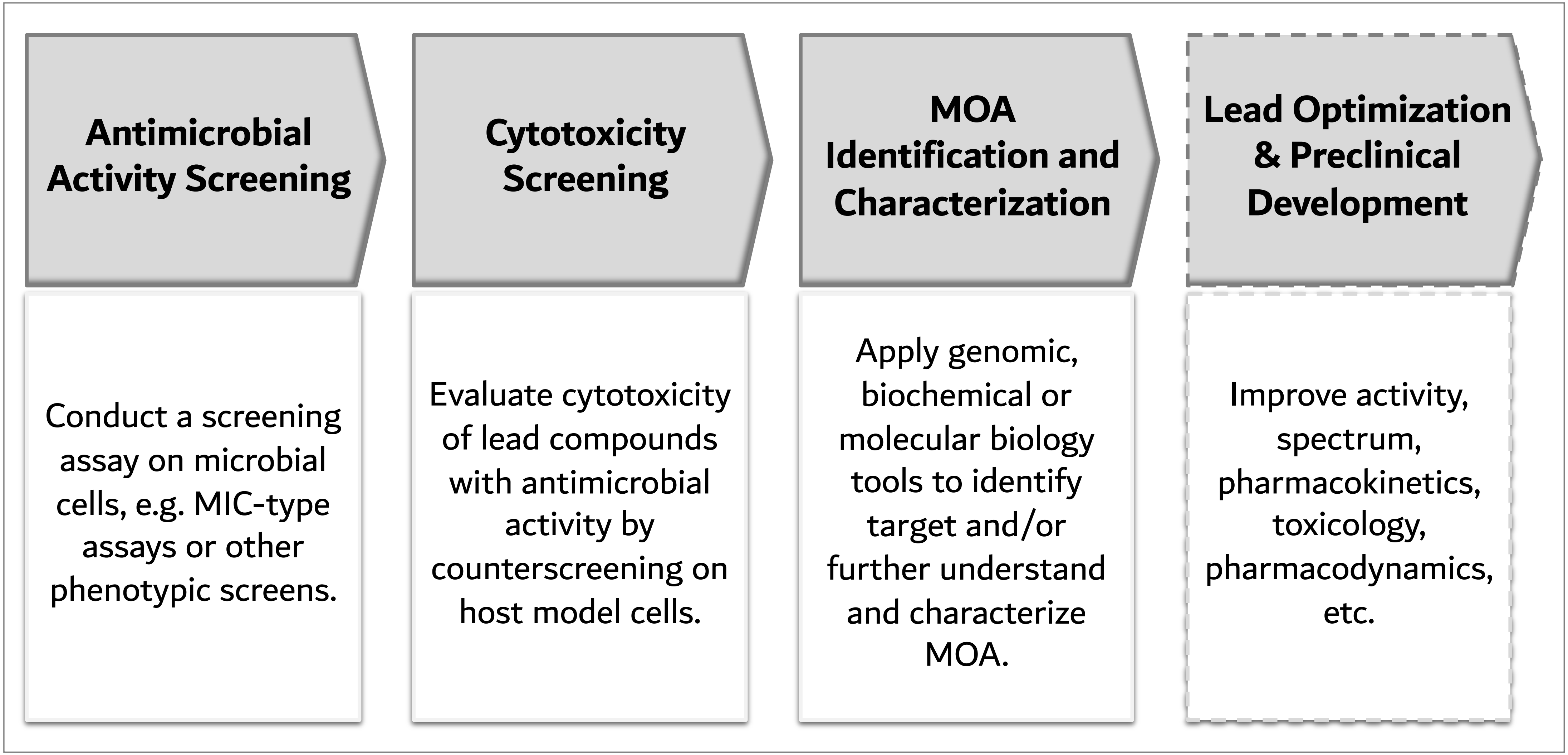
Antimicrobial Activity Screening
Conduct a screening assay on microbial cells, e.g. MIC-type assays or other phenotypic screens.
Cytotoxicity Screening
Evaluate cytotoxicity of lead compounds with antimicrobial activity by counterscreening on host model cells.
MOA Identification and Characterization
Apply genomic, biochemical or molecular biology tools to identify target and/or further understand and characterize MOA.
Lead Optimization & Preclinical Development
Improve activity, spectrum, pharmacokinetics, toxicology, pharmacodynamics, etc.
