# Supplementary figures and images for: Antibiotic Discovery: Where Have We Come from, Where Do We Go?
Source: Antibiotics (Basel). 2019 Apr 24;8(2):45. doi: 10.3390/antibiotics8020045 (PMC6627412; doi:10.3390/antibiotics8020045)

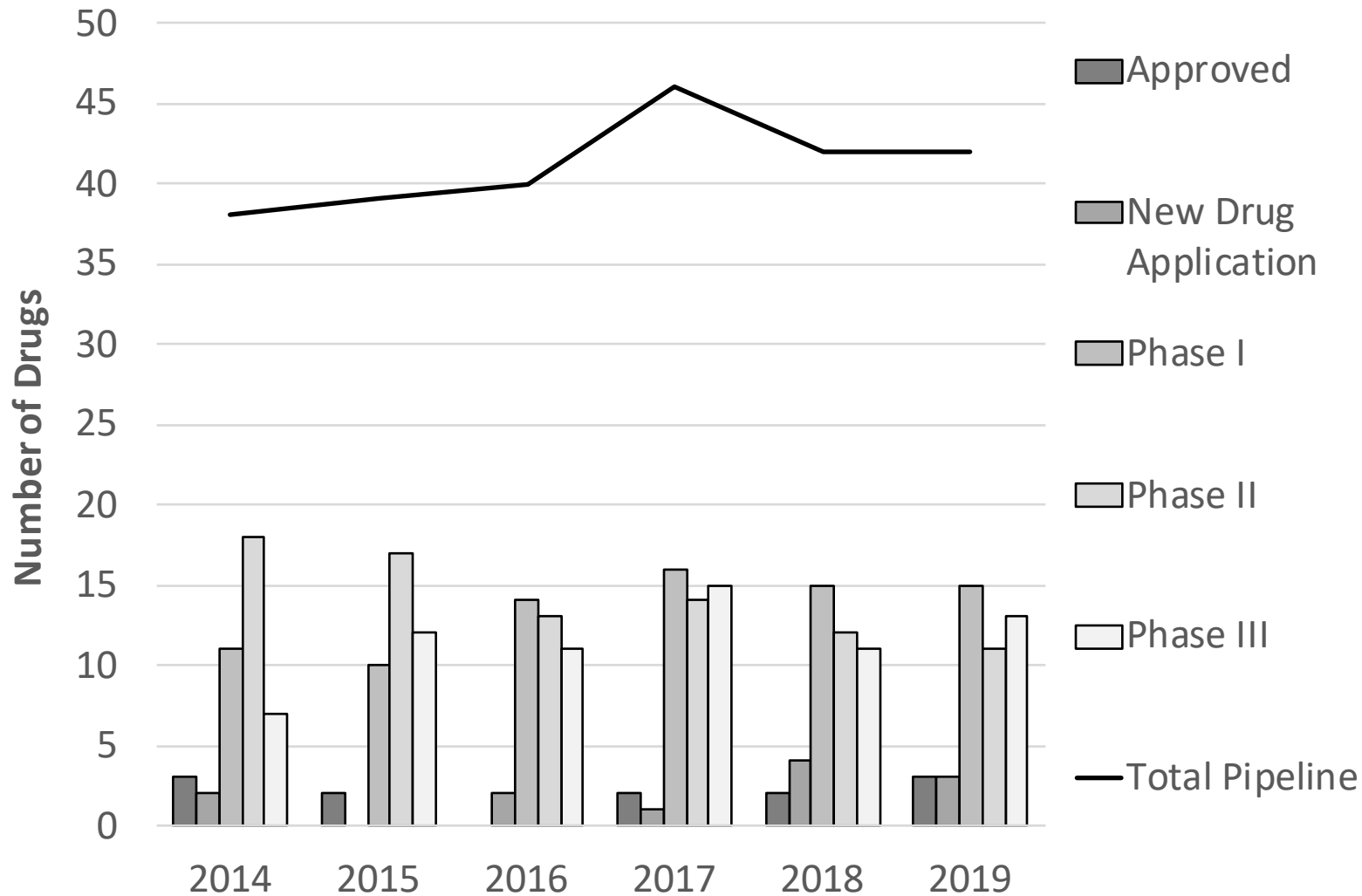

Supplement: Supplementary File 1 [file antibiotics-08-00045-s001.zip › AntibioticPipeline.pdf]
